# Determination of Pendimethalin Dynamic Residual Distribution in Crucian Carp Tissues and Associated Risk Assessment

**DOI:** 10.1155/2021/9984230

**Published:** 2021-07-02

**Authors:** Lu Qiao, Na Yuan, Gang Han, Bo Cheng, Defu Zhang, Jinlong Song, Yingchun Mu

**Affiliations:** ^1^Key Laboratory of Control of Quality and Safety for Aquatic Products (Ministry of Agriculture and Rural Affairs), Chinese Academy of Fishery Sciences, Beijing 100141, China; ^2^College of Food Science and Engineering, Bohai University, Jinzhou, Liaoning 121000, China

## Abstract

Pendimethalin has been considered a moderately to extremely toxic compound for fish and aquatic organism. This study developed the determination of dynamic residual distribution for pendimethalin in crucian carp tissues (muscle, liver, kidney, gill, and blood) under semistatic exposure system by high-performance liquid chromatography-tandem mass spectrometry (HPLC-MS/MS) method. The pendimethalin residues in various fish tissues increased initially and then decreased, and the residue amount of pendimethalin varied from tissue to tissue of crucian carp. Particularly, the pendimethalin accumulation in most fish tissues made significant decreases at two-time points. Pendimethalin was initially absorbed and enriched by fish body, and then partial pendimethalin was discharged into the outside environment through the metabolism function of crucian carp. The residue levels of pendimethalin distributed in crucian carp were ranked in the following decreasing order: liver > kidney > gill or muscle > blood, attributed to the fact that pendimethalin tends to accumulate in lipid-rich tissues of fish. Risk assessment results indicated that the chronic risk from dietary exposure to pendimethalin through crucian carp consumption for Chinese residents was acceptable, along with a lower estimated exposure dose (EED) than acceptable daily intake (ADI) and risk quotient (RQ) < 1. This study performed the first analysis for pendimethalin residual distribution in crucian carp tissues under semistatic exposure condition and provided a reference for pollution control and risk assessment of pendimethalin aimed at aquatic products.

## 1. Introduction

Pesticides, including an extensive amount of chemicals such as herbicides, insecticides, and fungicides, are commonly found in aquatic ecosystems [1]. Extensive use of pesticides in agriculture brings about a toxicological threat to environment, particularly the aquatic ecosystem, as they possess persistent nature [2]. Like all pesticides, herbicides cause some harmful effects on nontarget organisms as well as their benefits in agriculture, which are used on or near the soil, and in the water for aquatic weed control [3, 4]. Herbicides are applied worldwide with a consumption of about 47.5% among total pesticides [5]. Although being considered with low toxicity for mammals, herbicides can pose negative effects on fish even at low concentrations [6].

Belonging to a dinitroaniline herbicide active substance, pendimethalin (N-(1-ethylpropyl)-2, 6-dinitro-3,4-xylidine) is frequently applied in terrestrial systems, which selectively controls grassy weeds and certain broadleaf weeds in a variety of crops and in noncrop areas [7]. Pendimethalin has been on the market for approximately 30 years, predominantly used for soil as a preplant, preemergence, or sometimes postemergence herbicide [8]. Pendimethalin is registered for use in a large number of countries in every continent, each of which contains a range of growing conditions for pendimethalin utilization on crops. Due to the common usage of this chemical compound with various formulations, pendimethalin has been detected at high concentrations in rivers in Brittany, France (840 ng/L), and in surface waters in several European countries, which is known to affect various biotic components of freshwater ecosystems, such as phytoplankton, zooplankton, and aquatic fauna [9]. In Brazil, the contamination by pendimethalin in 53 river water samples ranged from 0.06 to 0.38 *μ*g/L [10]. The hard clam samples showed contamination by pendimethalin at levels from 16.1 to 60.1 ng/g in China [11]. The pendimethalin contamination reached the aquatic environments mainly via run-off from sprayed fields, leaching, evaporation, and drift [12]. Pendimethalin applied in agricultural fields entered into nearby aquatic systems, resulted in detrimental effects on nontarget aquatic organisms including fishes [13].

Pendimethalin has been categorized as a persistent bioaccumulative toxic (PBT) herbicide and a group C carcinogen (*possible human carcinogen*) [14]. It is considered a moderately to extremely toxic compound for fish and aquatic organism, which can generate long-lasting metabolites and induce deleterious effects on fishes [15]. Currently, some researches about the toxic impacts of pendimethalin on nontarget aquatic organisms such as fish have been published. The toxicity of pendimethalin varies from species to species and its effects on various aspects of fishes have been reported from time to time, covering alterations of biochemical parameters in *Oreochromis niloticus* [16], biochemical and histological perturbations in liver, kidney, and gill of freshwater fish [15], histological changes in the gill epithelium and altered general branchial functions in *Tilapia nilotica* [17], induction of oxidative stress in rainbow trout and *Channa punctatus* [7, 18], effect on the susceptibility of *Oncorhynchus mykiss* L. to viral hemorrhagic septicemia virus (VHSV) [1], and neurotoxicological assessment of pendimethalin in *Channa punctata* Bloch [2]. In order to resist these toxic impacts, fish must activate several responses to such environmental stressors and give rise to alter the fish metabolism including growth, reproduction, survival skills, and immunity.

The maximum residues limits (MRLs) for pendimethalin in different varieties of agricultural products such as grains and vegetables have been established by China, while lacking MRLs in aquatic products [19]. European Union (EU), the United States, and Japan have developed the MRLs for pendimethalin in aquatic products to strengthen the management of pesticides. The MRLs for pendimethalin are defined as 50 *μ*g/kg in crayfish (USA) and 300 *μ*g/kg in fish (Japan). Based on GB 2763-2019 in China, named as national food safety standard-maximum residue limits for pesticides in food, the acceptable daily intake (ADI) of pendimethalin is set as 100 *μ*g/kg bw.

In recent years, the effects of pendimethalin on the biotic components of freshwater ecosystems have been reported. However, few data about the residue and risk assessment of pendimethalin in fish are available. In view of the migration and bioconcentration of compounds from the environment, it is necessary to determine the residue levels of pendimethalin in aquatic products, which are identified as the main aquatic pathway for pendimethalin pollutants to be transferred into human body. Thus, the residue and risk assessment of pendimethalin in crucian carp were performed in this study, for that crucian carp is one of the most important edible fishes in China. The typical method of high-performance liquid chromatography-tandem mass spectrometry (HPLC-MS/MS) was developed for pendimethalin determination in crucian carp. The analysis for dynamic residual distribution of pendimethalin in different crucian carp tissues was carried out, and the risk assessment of dietary exposure to pendimethalin through crucian carp consumption for Chinese residents was implemented. This study provided a reference for pendimethalin pollution control and risk assessment aimed at aquatic products.

## 2. Materials and Methods

### 2.1. Reagents and Materials

The standard pendimethalin (purity 97.34%) was purchased from Dr. Ehrenstorfer GmbH (Augsburg, Germany). Methanol and acetonitrile of chromatographic grade and acetic acid of mass spectrometric grade were acquired from Thermo Fisher Scientific (USA). Both magnesium sulfate anhydrous (MgSO_4_) and sodium chloride (NaCl) of analytical grade were purchased from Sinopharm Chemical Reagent Co., Ltd. (Shanghai, China). The sorbent of primary and secondary amine (PSA) with guaranteed reagent was obtained from CNW Technologies GmbH (Dusseldorf, Germany). In addition, the ultrapure water applied during the experiments was acquired from Watsons.

A desired number of crucian carps with a similar size of (17 ± 1) cm × (6 ± 1) cm and weight of (140 ± 20) g obtained from local aquafarm were transported to the laboratory and placed in an aquarium being cleaned and disinfected. The experimental crucian carps were acclimated for one week in the aquarium (97.5 cm × 47.5 cm × 45 cm) and fed with feedstuff at 3 percent of body weight once a day. The water used for aquaculture was the tap water being stored and dechlorinated for more than 24 hours. The dissolved oxygen in water was maintained to be higher than 8.0 mg/L by continuous oxygen filling. The temperature of the water was 28 ± 1°C and pH was 6.5 ± 0.2, along with the total hardness of 28 ± 0.05 mg/L, dissolved oxygen of 6.6 ± 0.01 mg/L, and salinity of 0.6 ± 0.01 ng/L. The water was changed once a day. After temporary feeding for one week, healthy and active crucian carps with regular size were selected for further experiment.

### 2.2. Experiment Conditions

After acclimatization, the selected healthy and active crucian carps with regular size (140 ± 20 g) were exposed to medicated bath water (40 L) with pendimethalin of 13.8 *μ*g/L and 41.4 *μ*g/L in a semistatic system. Each treatment group contained crucian carp of 18 tails. Three replicates were set for each treatment group, and the blank control group was set at the same time. The crucian carps were fed with feedstuff at 1.5 percent of body weight once a day. To maintain the hygienic environment, fecal matter and other waste materials were removed in time during the exposure experiment.

The crucian carps with a number of 180 tails were exposed to bath doses of 13.8 *μ*g/L and 41.4 *μ*g/L pendimethalin for 8 consecutive days. According to GB/T 21858-2008 (Chemicals-Bioconcentration-Semistatic fish test) and GB/T 31270.7-2014 (Test guidelines on environmental safety assessment for chemical pesticides-Part 7: Bioconcentration test), the crucian carps were exposed to 10% of the 96 h LC_50_ pendimethalin (138 *μ*g/L) in semistatic test [20, 21]. Besides, through investigation, it was found that the pendimethalin concentration of 41.4 *μ*g/L was generally used by farmers. Thus, the concentrations of 13.8 μg/L and 41.4 μg/L for pendimethalin were selected in this work. Water samples were collected from aquaculture water bath at 0 h, 6 h, 24 h, 48 h, 96 h, 144 h, and 192 h in each treatment group and stored at −80°C. Different tissues of crucian carps were collected from three fishes at time points including 4 d, 6 d, 8 d, 9 d, 10 d, 12 d, 15 d, 18 d, 23 d, 28 d, and 33 d. The blood samples were acquired from the caudal vein using the heparinized plastic syringe for each experimental crucian carp, and thereafter, muscle with skin, gill filament, liver, and kidney were excised from each crucian carp.

### 2.3. Sample Preparation

Each water sample of 200 mL was processed by a 0.45 *μ*m filter membrane and then added to the solid phase extraction (SPE) cartridge of hydrophile-lipophile balance (HLB) with a speed of 6 mL/min. After elution with an 8 mL mixture of dichloromethane and acetone (v:v = 1 : 1) and drying under nitrogen gas flow, the sample to be tested was adjusted to a constant volume of 1 mL using the initial mobile phase. Finally, the water sample after treatment by a 0.22 *μ*m nylon membrane was determined using HPLC-MS/MS method.

Minced muscle, gill, liver, and kidney of crucian carp obtained by the surgical scissors were labeled and refrigerated at −80°C. The crucian carp tissue samples (2 g) after homogenization treatment were transferred to a 25 mL polypropylene (PP) centrifuge tube, along with 0.6 g NaCl and 10 mL acetonitrile being added. After vortex mixing for 30 s, ultrasonic treatment for 20 min, and centrifugation for 5 min at 4000 r/min, the upper organic phase of 8 mL was transferred to a centrifuge tube loaded with 0.2 g PSA and 0.4 g MgSO_4_. Subsequently, the mixture solution was with vortex mixing for 30 s and centrifuged at 4000 r/min for 5 min. Then the supernatant of 5 mL was processed with nitrogen blowing, followed by being redissolved with 1 mL initial mobile phase and filtered through a 0.22 *μ*m nylon membrane. Finally, the pendimethalin residue concentration in different tissues of crucian carp was detected by HPLC-MS/MS method.

### 2.4. Instrumental Analysis

An Accela-TSQ Quantum Access Max triple-quadrupole mass spectrometer instrument (Thermo Fisher Scientific, USA) was used for the detection of pendimethalin concentration. A Thermo Hypersil GOLD C18 column (150 mm × 2.1 mm, 5 *μ*m) was used for the chromatographic separation of pendimethalin. LC was carried out using a gradient elution procedure under the column temperature of 40°C with a flow rate of 0.3 mL/min and injection volume of 10 *μ*L. The binary mobile phase was comprised of aqueous solution containing 0.1% (v/v) acetic acid (eluent A) and methanol solution containing 0.1% (v/v) acetic acid (eluent B). The gradient elution mode started from 5% A and 95% B until 8 min. Then mobile phase A was increased to 80% at 8 min and continuously increased to 100% at 12 min. Correspondingly, mobile phase B was reduced to 20% at 8 min and reduced to 0% at 12 min (seen in Table 1). The MS scanning was performed using selected reaction monitoring (SRM) mode under positive ionization condition, with ionspray voltage of 3500 V, ion source temperature of 325°C, sheath gas velocity of 35 arb, and auxiliary gas velocity of 8 arb. The monitoring ion pairs consisted of quantitative ion pair with m/z of 282.1/211.9 and qualitative ion pair with m/z of 282.1/193.9 and 282.1/250.2.

### 2.5. Method Validation

The developed technique was validated as a quantitative confirmatory method to evaluate its viability, covering the performance parameters of linearity, accuracy, precision, limit of detection (LOD), and limit of quantitation (LOQ) [22, 23]. Linearity of the HPLC-MS/MS method for pendimethalin detection was assessed by calibration curve at six concentration levels of 2 ng/mL, 5 ng/mL, 10 ng/mL, 20 ng/mL, 50 ng/mL, and 100 ng/mL. The accuracy was estimated by recovery as percentage of measured pendimethalin concentration versus spiked concentration. The precision was expressed by determining the relative standard deviation (RSD) at 10 ng/mL pendimethalin. The LOD and LOQ were calculated as three and ten times of signal-to-noise (S/N) ratio, respectively. The sample concentration lower than its corresponding LOQ was considered to be undetectable [24].

### 2.6. Dietary Exposure Risk Assessment

The consumption of food contaminated with pendimethalin is the main route for human exposure to pendimethalin. Currently, the MRLs for pendimethalin in aquatic products have not been established in China, while ADI value has been recommended by GB 2763-2019. According to relevant risk assessment references of pesticides [25–28], the related estimated exposure dose (EED) and risk quotient (RQ) of pendimethalin were measured to evaluate the extent of exposure to pendimethalin residues through crucian carps for Chinese residents. The ADI recommended by GB 2763-2019 for pendimethalin aimed at Chinese residents (100 *μ*g/kg bw) was employed for comparison. EED and RQ were calculated as follows:

(1) $$\begin{array}{c} \text{EED}\left(\mu \text{g}/\text{kg}\text{ bw}\right)=\text{RL}\left(\mu \text{g}/\text{kg}\right)\times \text{FI}\left(\text{kg}\right)÷\text{bw}\left(\text{kg}\right),\\ \text{RQ}=\text{EED}\left(\mu \text{g}/\text{kg}\text{ bw}\right)÷\text{ADI}\left(\mu \text{g}/\text{kg}\text{ bw}\right), \end{array}$$

where RL is residue level of pendimethalin, FI is food intake, and bw is body weight.

## 3. Results and Discussion

### 3.1. HPLC-MS/MS Method Validation

**Figure 1:**
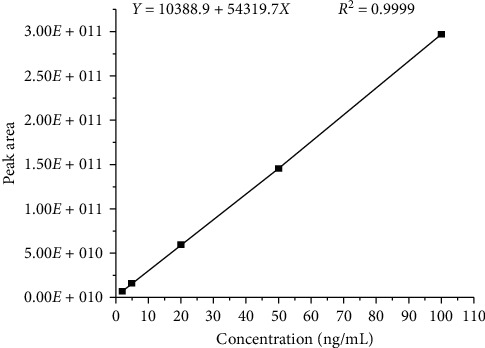
The calibration curve for pendimethalin detection by developed HPLC-MS/MS method.

Linearity of the present HPLC-MS/MS method for pendimethalin was determined in concentrations ranging from 2 ng/mL to 100 ng/mL. The calibration curves both in the solvent standard solution and in the matrix standard solution of fish tissues showed good linear relationships, with coefficients of determination (*R*^2^) > 0.99 (seen in Figure 1 and Table 2). The LOD and LOQ of this method for pendimethalin were estimated to be 0.2 *μ*g/kg and 0.6 *μ*g/kg, which met the requirement for residue analysis. In this study, pendimethalin at a concentration of 10 ng/mL was spiked into the blank tissue samples of crucian carp with three replicates to determine the accuracy and precision by intraday variability. As displayed in Table 2, the recoveries (*n* = 3) of pendimethalin in different tissues of crucian carp ranged from 87.89% to 117.64% with RSDs of 0.74%–2.22%. The results indicated that the developed method exhibited satisfactory accuracy and precision, among which all data were within the acceptable range.

### 3.2. Pendimethalin Residue in Aquaculture Water at Different Time Points

The crucian carps were exposed to pendimethalin at 13.8 *μ*g/L and 41.4 *μ*g/L in a semistatic system.  Table 3 showed the pendimethalin residue in aquaculture water at different time points. Under exposure concentration of 13.8 *μ*g/L, the residue level of pendimethalin in water with 13.14 *μ*g/L at 0 h presented dynamic changes, followed by a step decrease of pendimethalin residue from 0 h to 6 h to 12.47 *μ*g/L, from 6 h to 12 h to 7.82 *μ*g/L, from 12 h to 24 h to 3.71 *μ*g/L, and from 24 h to 48 h to 3.19 *μ*g/L. Then the pendimethalin residue was increased to 3.56 *μ*g/L from 48 h to 96 h and to 9.62 *μ*g/L from 96 h to 144 h. Finally, it was decreased to 3.38 *μ*g/L from 144 h to 192 h. The residual changes of pendimethalin in water were consistent under exposure concentrations of 41.4 *μ*g/L and 13.8 *μ*g/L. In conclusion, the pendimethalin residue in aquaculture water showed the change trends as follows: decrease from 0 h to 48 h, increase from 48 h to 144 h, and then decrease from 144 h to 192 h. The pendimethalin in water was absorbed and enriched by crucian carp, resulted in a decrease in pendimethalin residue from 0 h to 48 h. Then partial pendimethalin was discharged into aquaculture water through the gastrointestinal emptying and biological action of crucian carp, which lead to a residual increase of pendimethalin in water from 48 h to 144 h.

### 3.3. Residual Distribution of Pendimethalin in Different Tissues of Crucian Carp

**Figure 2:**
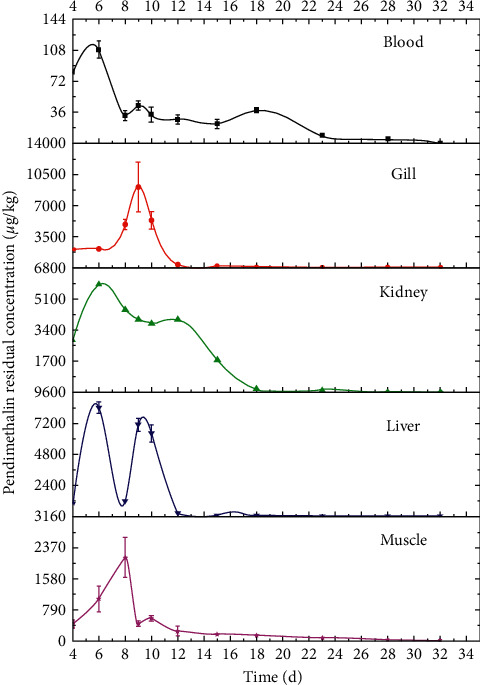
Dynamic residual distribution of pendimethalin in different tissues of crucian carp under 13.8 *μ*g/L exposure concentration.

**Figure 3:**
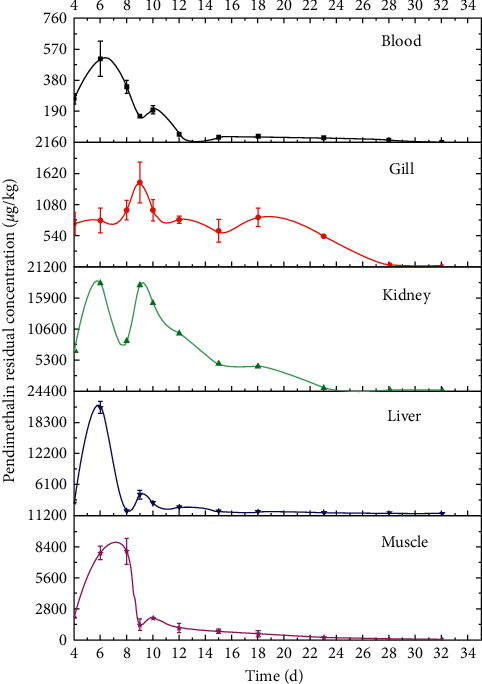
Dynamic residual distribution of pendimethalin in different tissues of crucian carp under 41.4 *μ*g/L exposure concentration.

The analysis for pendimethalin residual distribution in different tissues of crucian carp was performed in this study. Figures 2 and 3 showed the dynamic residual distribution of pendimethalin in crucian carp tissues at different time points of 4 d, 6 d, 8 d, 9 d, 10 d, 12 d, 15 d, 18 d, 23 d, 28 d, and 33 d. As displayed in Figures 2 and 3, pendimethalin was detected in muscle, liver, kidney, gill, and blood of crucian carps under both 13.8 *μ*g/L and 41.4 *μ*g/L exposure concentrations. In general, the residues of pendimethalin in each tissue of crucian carps were not uniformly regular. The pendimethalin distribution in fish gill versus time initially increased and sequentially decreased from the 9th day, generated by exposure to pendimethalin for 8 consecutive days. The pendimethalin residues in muscle, liver, kidney, and blood presented multiple concentration peaks. An abrupt significant decrease of pendimethalin residue in liver, kidney, and blood occurred on the 6th day, with residue concentration of 8424.47 and 21195.10 *μ*g/kg (liver), 5903.66 and 18346.39 *μ*g/kg (kidney), and 108.49 and 509.77 *μ*g/kg (blood) under 13.8 *μ*g/L and 41.4 *μ*g/L exposure concentrations, respectively. The pendimethalin residue in fish muscle presented a dramatic decline on the 8th day, with a residue of 2121.90 and 7969.23 *μ*g/kg under exposure concentrations of 13.8 *μ*g/L and 41.4 *μ*g/L. The second decrease of pendimethalin residue in liver, kidney, blood, and muscle occurred at different time points.

Pendimethalin herbicide accumulated in crucian carp with concentrations in the following decreasing order: liver > kidney > gill > muscle > blood under 13.8 *μ*g/L exposure concentration and liver > kidney > muscle > gill > blood under 41.4 *μ*g/L exposure concentration. In conclusion, the pendimethalin residue in liver was higher than that in other tissues, and the blood still presented the lowest residue accumulation. The results indicated that pendimethalin tends to accumulate in lipid-rich tissues. In addition, the decreases of pendimethalin residue in various tissues of crucian carp were resulted from the metabolism function of the fish body to discharge pendimethalin into the outside environment through feces and urine.

In China, rice-fish coculture was a common farming pattern, and pendimethalin, as a herbicide, was widely used in crops. Then pendimethalin residue occurred in nontarget aquatic organisms including fishes. The exposure concentration of 13.8 *μ*g/L was sometimes used in the rice-fish coculture system, while 41.4 *μ*g/L exposure concentration was rarely used. Currently, China has not set the MRLs for pendimethalin in aquatic products. The MRLs for pendimethalin are defined as 50 *μ*g/kg in crayfish (USA) and 300 *μ*g/kg in fish (Japan). Under 13.8 *μ*g/L exposure concentration, the pendimethalin residue in fish muscle was reduced below 300 *μ*g/kg on the 12th day. The results provided an important data foundation for pendimethalin control in the rice-fish coculture system. Further study for pendimethalin residue in other aquatic organisms needs to be performed, for that rice-crab coculture and rice-shrimp coculture are also common farming patterns in China.

### 3.4. Risk Assessment for Pendimethalin from Dietary Intake

EED and RQ were introduced for the risk assessment of pendimethalin from dietary intake, among which aquatic product was the only residual exposure route for pendimethalin. According to the data from China Statistical Yearbook 2019 by China's National Bureau of Statistics (NBS), per capita consumption of per year for aquatic products by urban residents was 14.8 kg in 2017 and 14.3 kg in 2018, while per capita consumption by national residents was 11.5 kg in 2017 and 11.4 kg in 2018. In line with the principle of risk maximization, per capita consumption per year for aquatic products of 14.8 kg was used to calculate the EED and RQ. In other words, the daily consumption of aquatic products was identified as 40.55 g. Combined with adult's body weight of 64.1 kg from the National Physical Fitness Monitoring Bulletin by General Administration of Sport of China, EED and RQ applied for risk assessment were obtained and tabulated.

The ADI of pendimethalin was stipulated to 100 *μ*g/kg bw by GB 2763-2019. Under 13.8 and 41.4 *μ*g/L exposure concentrations, the RQs for pendimethalin in different tissues of crucian carp ranged from 0.0125 × 10^−3^ to 7.47 × 10^−2^ and 0.06 × 10^−3^ to 14.10 × 10^−2^, with EEDs of 0.00125–5.61 *μ*g/kg bw and 0.006–14.10 *μ*g/kg bw (seen in Tables 4 and 5). The pendimethalin EEDs of all fish samples were lower than 100 *μ*g/kg bw and RQ values were lower than 1, indicating that the chronic risk of pendimethalin was acceptable. However, pendimethalin is registered for use in a variety of agricultural products, and the dietary structure of Chinese residents is diverse and complex. Residues of pendimethalin may be present in different agricultural products, resulting in the increase of exposure to pendimethalin through total dietary to some extent. Thus, the risk of pendimethalin exposure for Chinese residents may be greater than this estimated level, and a daily dose of harmful intakes from total dietary may affect public health in the long term.

## 4. Conclusions

In this study, the residue distribution determination of pendimethalin in different tissues of crucian carps was performed by HPLC-MS/MS. The developed method exhibited satisfactory parameters of high linearity, accuracy, and precision as well as low LOD and LOQ. In semistatic test, the pendimethalin residues were detected in muscle, liver, kidney, gill, and blood of crucian carps, and the residue amount of pendimethalin varied from tissue to tissue of crucian carp. Residue levels of pendimethalin in various fish tissues increased initially and then decreased gradually, and the accumulation of pendimethalin in most tissues made significant decreases at two-time points. The results indicated that pendimethalin was absorbed and enriched by the fish body, and then partial pendimethalin was discharged into the outside environment through feces and urine by the metabolism function of fish. Furthermore, the liver presented the highest pendimethalin residue among all detected tissues of crucian carps, attributed to the fact that pendimethalin tends to accumulate in lipid-rich tissues. In addition, risk assessment results demonstrated that dietary exposure to pendimethalin through crucian carp consumption for Chinese residents was lower than the acceptable ADI level of 100 *μ*g/kg bw, along with RQ < 1. Further studies are necessary to assess the resident exposure to pendimethalin residues and their health risk associated with the consumption of total dietary.

## Figures and Tables

**Table 1:** The gradient elution procedure for pendimethalin determination by HPLC-MS/MS.

Time (min)	Flow rate (mL/min)	Mobile phase A (%)	Mobile phase B (%)
0	0.3	5	95
8	0.3	80	20
12	0.3	100	0

**Table 2:** The calibration curves, recoveries, and RSDs of pendimethalin in different tissues of crucian carp.

Tissue of crucian carp	Linear equation	*R* ^2^	Recovery (%)	RSD (%)
Muscle	*Y* = -8083.02 + 18061.1*X*	0.9995	87.89	1.52
Gill	*Y* = -8730.23 + 16067.2*X*	0.9987	111.59	2.22
Liver	*Y* = 6050.18 + 16858.5*X*	0.9996	90.79	1.71
Kidney	*Y* = -36935.5 + 18892.9*X*	0.9970	117.64	0.74
Blood	*Y* = -33251.7 + 21037.3*X*	0.9977	96.49	1.14

**Table 3:** Pendimethalin residues in aquaculture water for crucian carp under different exposure concentrations of pendimethalin.

Sample time for aquaculture water (h)	Exposure concentration of 13.8 *μ*g/L pendimethalin		Exposure concentration of 41.4 *μ*g/L pendimethalin	
	Residue concentration (*μ*g/L)	RSD (%)	Residue concentration (*μ*g/L)	RSD (%)
0	13.14	3.34	39.51	3.59
6	12.47	0.61	16.99	1.38
12	7.82	2.34	10.99	2.33
24	3.71	1.51	4.08	0.76
48	3.19	1.47	3.31	0.57
96	3.56	3.03	11.65	4.22
144	9.62	4.13	14.81	6.26
192	3.38	1.45	4.86	1.79

**Table 4:** Residues, EEDs, and RQs of pendimethalin in different tissues of crucian carp under 13.8 *μ*g/L exposure concentration.

Time (d)	Muscle			Liver			Kidney			Gill			Blood		
	Residue (*μ*g/kg)	EED (*μ*g/kg bw)	RQ (×10^−3^)	Residue (*μ*g/kg)	EED (*μ*g/kg bw)	RQ (×10^−3^)	Residue (*μ*g/kg)	EED (*μ*g/kg bw)	RQ (×10^−2^)	Residue (*μ*g/kg)	EED (*μ*g/kg bw)	RQ (×10^−2^)	Residue (*μ*g/kg)	EED (*μ*g/kg bw)	RQ (×10^−3^)
0	<LOD	—	—	<LOD	—	—	<LOD	—	—	<LOD	—	—	<LOD	—	—
4	314.53–497.47	0.20–0.32	1.99–3.15	889.60–1034.64	0.56–0.66	5.63–6.55	2852.45	1.80	1.80	1903.60–2266.84	1.20–1.43	1.20–1.43	61.24–96.83	0.039–0.061	0.39–0.61
6	803.78–1434.68	0.51–0.91	5.08–9.08	7970.72–8868.64	5.04–5.61	50.42–56.10	5903.66	3.73	3.73	1919.85–2372.11	1.21–1.50	1.21–1.50	101.90–119.88	0.064–0.076	0.64–0.76
8	1539.96–2418.16	0.97–1.53	9.74–15.30	1158.50–1233.46	0.73–0.78	7.33–7.80	4532.58	2.87	2.87	4322.97–5467.20	2.73–3.46	2.73–3.46	26.86–38.41	0.017–0.024	0.17–0.24
9	366.01–490.31	0.23–0.31	2.32–3.10	6542.36–7524.74	4.14–4.76	41.39–47.60	3980.02	2.52	2.52	6235.11–11808.80	3.94–7.47	3.94–7.47	40.58–50.05	0.026–0.032	0.26–0.32
10	521.37–644.06	0.33–0.41	3.30–4.07	5970.04–7212.00	3.78–4.56	37.77–45.62	3752.24	2.37	2.37	4581.88–6491.93	2.90–4.11	2.90–4.11	23.45–39.35	0.015–0.025	0.15–0.25
12	158.45–389.94	0.10–0.25	1.00–2.47	260.48–288.20	0.17–0.18	1.65–1.82	3984.87	2.52	2.52	316.70–345.02	0.20–0.22	0.20–0.22	21.82–32.45	0.014–0.021	0.14–0.21
15	139.95–185.25	0.09–0.12	0.89–1.17	68.10–99.46	0.043–0.063	0.43–0.63	1754.05	1.11	1.11	113.09–238.58	0.07–0.15	0.07–0.15	18.06–28.21	0.011–0.018	0.11–0.18
18	135.80–143.74	0.086–0.091	0.86–0.91	48.37–67.15	0.031–0.042	0.31–0.42	184.38	0.12	0.12	70.83–111.27	0.04–0.07	0.04–0.07	34.88–39.99	0.022–0.025	0.22–0.25
23	62.94–94.09	0.04–0.06	0.40–0.60	38.40–41.94	0.024–0.027	0.24–0.27	130.51	0.08	0.08	32.46–46.49	0.02–0.03	0.02–0.03	6.75–10.64	0.004–0.007	0.04–0.07
28	22.50–27.68	0.014–0.018	0.14–0.18	23.33–24.11	0.0148–0.153	0.148–0.153	15.29	0.01	0.01	11.86–21.86	0.008–0.014	0.008–0.014	3.20–6.09	0.002–0.004	0.02–0.04
32	<LOD	—	—	1.98–2.03	0.00125–0.00128	0.0125–0.0128	<LOD	—	—	<LOD	—	—	<LOD	—	—

**Table 5:** Residues, EEDs, and RQs of pendimethalin in different tissues of crucian carp under 41.4 *μ*g/L exposure concentration.

Time (d)	Muscle			Liver			Kidney			Gill			Blood		
	Residue (*μ*g/kg)	EED (*μ*g/kg bw)	RQ (×10^−2^)	Residue (*μ*g/kg)	EED (*μ*g/kg bw)	RQ (×10^−2^)	Residue (*μ*g/kg)	EED (*μ*g/kg bw)	RQ (×10^−2^)	Residue (*μ*g/kg)	EED (*μ*g/kg bw)	RQ (×10^−3^)	Residue (*μ*g/kg)	EED (*μ*g/kg bw)	RQ (×10^−3^)
0	<LOD	—	—	<LOD	—	—	<LOD	—	—	<LOD	—	—	<LOD	—	—
4	1733.16–2218.43	1.10–1.40	1.10–1.40	2234.68–2620.34	1.41–1.66	1.41–1.66	6767.53	4.28	4.28	544.26–948.05	0.34–0.60	3.44–6.00	230.04–294.00	0.15–0.19	1.46–1.86
6	7295.44–8492.80	4.62–5.37	4.62–5.37	19985.70–22294.40	12.64–14.10	12.64–14.10	18346.39	11.61	11.61	574.65–993.67	0.36–0.63	3.64–6.29	405.00–617.62	0.26–0.39	2.56–3.91
8	6627.32–8717.58	4.19–5.51	4.19–5.51	749.80–998.70	0.47–0.63	0.47–0.63	8528.57	5.40	5.40	818.17–1149.01	0.52–0.73	5.18–7.27	292.78–367.52	0.19–0.23	1.85–2.32
9	774.32–1656.56	0.49–1.05	0.49–1.05	3134.96–4606.52	1.98–2.91	1.98–2.91	18018.77	11.40	11.40	1212.94–1863.20	0.77–1.18	7.67–11.79	152.94–166.94	0.10–0.11	0.97–1.06
10	1818.53–2068.84	1.15–1.31	1.15–1.31	2080.80–2772.42	1.32–1.75	1.32–1.75	15016.87	9.50	9.50	854.18–1192.08	0.54–0.75	5.40–7.54	174.04–223.44	0.11–0.14	1.10–1.41
12	812.22–1535.34	0.51–0.97	0.51–0.97	1303.02–1903.74	0.82–1.20	0.82–1.20	9803.11	6.20	6.20	741.18–846.77	0.47–0.54	4.69–5.36	38.40–51.51	0.02–0.03	0.24–0.33
15	580.83–946.64	0.37–0.60	0.37–0.60	785.44–859.68	0.50–0.54	0.50–0.54	4647.19	2.94	2.94	427.75–822.45	0.27–0.52	2.71–5.20	24.402–31.15	0.015–0.020	0.15–0.20
18	242.63–678.94	0.15–0.43	0.15–0.43	605.80–704.22	0.38–0.45	0.38–0.45	4243.22	2.68	2.68	756.19–1037.61	0.48–0.66	4.78–6.56	32.80–38.70	0.021–0.024	0.21–0.24
23	167.63–229.10	0.11–0.14	0.11–0.14	471.60–547.64	0.30–0.35	0.30–0.35	514.21	0.33	0.33	494.70–553.75	0.31–0.35	3.13–3.50	19.29–30.03	0.012–0.019	0.12–0.19
28	24.92–33.86	0.016–0.021	0.016–0.021	427.60–427.93	0.2705–0.2707	0.2705–0.2707	90.57	0.06	0.06	24.18–36.44	0.015–0.023	0.15–0.23	9.84–10.61	0.006–0.007	0.06–0.07
32	<LOD	—	—	278.24–286.08	0.176–0.181	0.176–0.181	<LOD	—	—	<LOD	—	—	<LOD	—	—

## Data Availability

The data used to support the findings of the present study are available from the corresponding author upon request.
